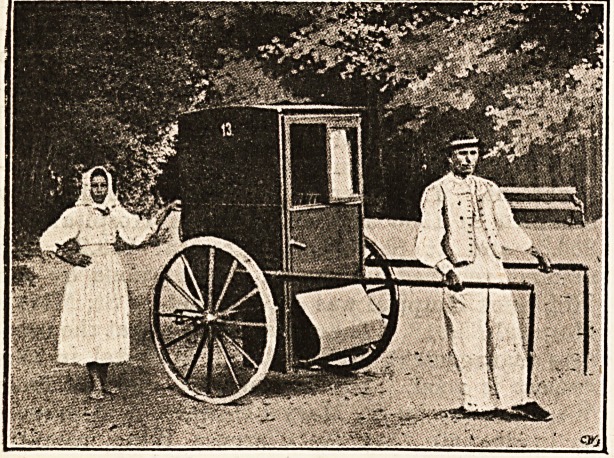# A Hungarian Health Resort

**Published:** 1907-06-08

**Authors:** 


### A HUNGARIAN HEALTH RESORT.

### THE MUD-BATHS OF PÖSTYEN.

Hungary is the land of the *cur* as well as the *csardas.* Here Nature has provided cures for well-nigh every ill that flesh is heir to, and the invalid in search of health may sample baths of all sorts—hot springs of sulphur, iron, magnesia, alum, mud—baths ranging from those of primitive simplicity known only to wanderers in the valleys of the Carpathians, to the renowned water of Hercules in Southern Hungary or the Kaiserbad of Buda-Pesth.

In search of pleasure rather than of a cure, we were guided to Pöstyen's miraculous mud-baths by effective posters displayed at the railway stations and showing a nude giant standing on a rocky height and casting away a crutch which, apparently, a course of Pöstyen treatment had rendered superfluous. We consulted Baedeker and discovered that this Hungarian Lourdes was less than four hours by rail from Buda-Pesth, and about the same distance from Vienna; that the baths belonged to Count Erdödy; and that as long ago as 1599 they were known to the Turkish invaders of Hungary.

So one evening when the sun was setting on the beautiful blue Carpathians and gilding the waters of the River Waag we were jolted in the omnibus over the long ill-kept road that leads from the railway station to the little town that has grown up around the baths. The market-place, with its open stalls and groups of peasants in picturesque costumes, the queer little shops with stores of peasant embroideries, and the typically Magyar character and colour of the place, promised ample material for pen and pencil.

### The Patrons of Pöstyen.

Though it was late September and the summer season was well-nigh over, there were many victims of gout and rheumatism and other ills tottering about the streets and parks on crutches which they hoped to bequeath on their departure to the little museum of be-ribboned mementoes which we saw later in the entrance to one of the bath-houses. Most of the visitors were Hungarians, but a sprinkling of Austrians, Bohemians, Germans, and some of the many minor races that dwell in Hungary made up a polyglot company. Now and then, we were told, an English or American visitor will find his way to Pöstyen, following the example of the late Sir Spencer Wells, who, struck by a cure the Pöstyen mud had effected on the wounded hand of a Viennese surgeon of his acquaintance, visited the place after a meeting of the Hygienic Congress at Vienna which he had been attending. Having sampled the baths, Sir Spencer prophesied that one day Pöstyen would become a formidable rival to Vichy, Homburg, Carlsbad, and other well-known spas. But that day has not yet come, and we found the place delightfully free from the tourist element.

The bath-houses are built on a picturesque little island in the middle of the River Waag, amid shady beech groves where white peacocks, indigenous to the place, add a beauty to the scene. On the bridge and all about in the leafy avenues, as we made the tour of the baths with Herr Winter, the director, we came on invalids driving in quaint little carriages something between a rickshaw and a sedan chair, each drawn by a sturdy barefoot peasant man or woman in the picturesque costume of the district, the men in wide linen trousers and tunics, the women in short accordion-pleated skirts, blue aprons, and white short-sleeved bodices. Each villa has its own equipment of these little carriages and of "Infanterists," as the peasants who draw them are called.

### A Sliding Scale for Patients.

Provision is made for all classes, the poor peasant being able to have his bath of hot sulphur water or mud at certain hours for a few pence, while in the luxurious new "Franz-Josef bath-house" royal visitors are catered for in a special "Princes' bath," which has already earned its name from visits of Prince Ferdinand of Bulgaria, Princess Pauline of Wurtemberg, and other royal sufferers from gout or rheumatism. There are "Spiegel" or mirror baths for men only, others for ladies, and one for mixed bathing, besides innumerable separate baths fitted, some with wooden tubs, others with marble tanks, and many with duplicate bath-tubs, one for the hot mud, the other for the subsequent immersion in hot sulphur water. A bath costs from 1½ to 7 crowns (1s. 3d. to 6s. 5d.), according to the hour and class of bath. The new bath-house boasts a fine swimming-bath decorated in Moorish style, and the use of peasant embroideries in the *portières* adds an artistic charm to a mud-bath in their neighbourhood. All the baths are fitted with double supply pipes, one for the water at 60 degrees centigrade—its temperature when it is drawn from the river—the other for the same water artificially cooled in accordance with the doctor's instructions. That the mud which the bare-legged men in blue tunics were drawing from beneath the cool surface-waters of the river was hot and not merely tepid we proved for ourselves by dipping our fingers into a panful as it was drawn up. It was like hot porridge, and the water, smelling strongly of sulphur, which we sampled in the well-house later came up so hot that it needed a dash of cold water before we could sip it. Recently the hot mud has been found to contain radium, and this discovery may bring about the fulfilment of Sir Spencer Wells's prophecy and make the place a Mecca of modern medicine. Meanwhile it certainly seems to work wonders for those who suffer from any form of rheumatism, gout, neuralgia, sciatica, and lumbago. We could see patients growing daily more independent of their crutches, and a Hungarian officer whom we met told us he had come there a helpless cripple, and after five weeks' treatment was quite cured and was staying on for pleasure. There is, by the way, special provision in a military institute for officers both of the Hungarian and the Austrian armies, and a variety of gay uniforms mingled in the crowd that mustered in the park to listen to the gipsy band, without which no Hungarian open-air resort is complete.

### The Mud-baths.

Curiosity made us sample the mud-baths, and we were thus initiated into rites that involved the services of a couple of blue-bloused Hungarians, who brought the hot mud to our cabins in wooden creels, swung on poles, and of a buxom bath-woman who, wrapping us in blankets, left us to soak our limbs in the mud for twenty minutes, timing herself by a "facing both ways" alarm clock, which brought her to the minute to supervise the later stages of the ceremony. These included a plunge in hot sulphur water, a rub-down with warm linen, and a siesta on a couch in our respective cabins. Perspiration plays its part in the cure, and our resolve to walk home instead of taking a rickshaw caused consternation to our bath-lady, and doubtless branded us in her esteem as "mad Angols."

A casino in the park, with reading-room, library, music-rooms, and summer theatre, provides the usual amusements of spa life. But our favourite entertainment was to saunter about the little town studying peasant life and buying peasant embroideries, worked in winter by the women of the district when field work ceases. Every day would bring its own picture—now a religious procession of half a mile or so of flower-crowned girls, each with a lighted taper, chanting hymns, on their way to some shrine; another day a string of wagons overflowing with peasants, all in red and russet, singing gaily on their way to some hillside vintage. Sundays and *fête* days brought out the peasants in wonderful gala dress to the wayside inns to dance the *csardas* to their own wonderful music or to polka to the tune of "John Brown's Body" or the last American cake-walk played by gipsy fiddlers brown as berries. The colours that blazed in the kerchiefs and hair-ribbons, the bodices and aprons and short pleated skirts, were suggestive of a broken rainbow. And always for background were the distant Carpathian mountains and the fascinating River Waag, with the great timber rafts rushing down stream, and on the banks women wading knee-deep to wash their linen *à l'écossaise*.

### WHERE TO STAY.

Besides modest hotels, where board may be had for as little as five crowns (4s. 7d.) a day, and villas with pleasant gardens, such as the villa reherek, where we were comfortably quartered, there is in the Cur Park itself a resplendent new hotel, the Grand Hotel Ronai, which in comfort and elegance can vie with the Carlton or the Ritz. Steam-heated, with electric-light, a motor omnibus, an excellent cuisine, and balconies commanding beautiful views of the mountains and river, this hotel may do more than anything else to bring English and American visitors to the Hungarian Lourdes, and so fulfil the prophecy of Sir Spencer Wells, who saw in Pöstyen a fine field for some enterprising financier or syndicate with the capital needed to develop its resources and make its miracle-working mud-springs known to the whole world.

## Figures and Tables

**Figure f1:**